# Impact of endometriosis on obstetric outcome after natural conception: a multicenter Italian study

**DOI:** 10.1007/s00404-021-06243-z

**Published:** 2021-10-08

**Authors:** N. Berlanda, W. Alio, S. Angioni, V. Bergamini, C. Bonin, P. Boracchi, M. Candiani, G. Centini, M. N. D’Alterio, S. Del Forno, A. Donati, D. Dridi, D. Incandela, L. Lazzeri, A. Maiorana, A. Mattei, J. Ottolina, A. Orenti, A. Perandini, F. Perelli, I. Piacenti, I. Pino, M. G. Porpora, S. Scaramuzzino, R. Seracchioli, E. Solima, E. Somigliana, R. Venturella, P. Vercellini, P. Viganò, M. Vignali, F. Zullo, E. Zupi, Endometriosis Treatment Italian Club (ETIC)

**Affiliations:** 1 Department of Obstetrics and Gynecology, Fondazione IRCCS Ca’ Granda Ospedale Maggiore Policlinico, Università degli Studi di Milano, Via della Commenda 12, 20122 Milan, Italy; 2 Department of Obstetrics and Gynecology, Ospedale Civico, Piazza Nicola Leotta 4, 90127 Palermo, Italy; 3 Department of Surgical Sciences, Università di Cagliari, Cittadella Universitaria, 09042 Cagliari, Italy; 4 Azienda Ospedaliera Universitaria Integrata, Università di Verona, Piazzale A. Stefani 1, 37126 Verona, Italy; 5 Department of Clinical Sciences and Community Health, Laboratory of Medical Statistics, Epidemiology and Biometry “G. A. Maccacaro”, Università di Milano, Via Vanzetti 5, 20133 Milan, Italy; 6 Department of Obstetrics and Gynecology, IRCCS San Raffaele Scientific Institute, Via Olgettina 60, 20132 Milan, Italy; 7 Department of Gynecology, Università di Siena, Siena, Italy; 8 Department of Molecular and Developmental Medicine, Università di Siena, Strada delle Scotte 4, 53100 Siena, Italy; 9 Gynaecology and Human Reproduction Physiopathology Unit, Department of Medical and Surgical Sciences, DIMEC, Sant’Orsola Hospital, Università di Bologna, Via Massarenti 13, 40138 Bologna, Italy; 10 Division of Gynaecology and Obstetrics, Santa Maria Annunziata Hospital, USL Toscana Centro, Via Antella 58, 50012 Florence, Italy; 11 Department of Maternal and Child Health and Urology, Università di Roma La Sapienza, Policlinico Umberto I, Viale del Policlinico 155, 00161 Rome, Italy; 12 Preventive Gynecology Unit, European Institute of Oncology IRCCS, Via Ripamonti 435, 20141 Milan, Italy; 13 Department of Obstetrics and Gynecology, Macedonio Melloni Hospital, Milan, Italy; 14 Department of Biomedical Sciences for Health, Università degli Studi di Milano, Via Melloni 52, 20129 Milan, Italy; 15 Department of Clinical and Experimental Medicine, Magna Graecia University of Catanzaro, Viale Europa, 88100 Catanzaro, Italy; 16 Division of Genetics and Cell Biology, IRCCS San Raffaele Scientific Institute, Via Olgettina 60, 20132 Milan, Italy; 17 Department of Neuroscience, Reproductive Sciences and Dentistry, School of Medicine, Università di Napoli Federico II, Via Pansini 5, 80131 Naples, Italy

**Keywords:** Endometriosis, Obstetric complications, Preterm delivery, Adenomyosis, Placenta, Previa, Cesarean delivery

## Abstract

**Purpose:**

To evaluate obstetric outcome in women with endometriosis who conceive naturally and receive standard obstetric care in Italy.

**Methods:**

Cases were consecutive women with endometriosis managed in eleven Italian referral centers. Controls were women in whom endometriosis was excluded. All women filled in a questionnaire addressing previous natural pregnancies. Marginal logistic regression models were fitted to evaluate the impact of endometriosis on obstetric outcome. A post hoc analysis was performed within the endometriosis group comparing women with severe adenomyosis versus women with absent or mild adenomyosis.

**Results:**

Three hundred and fifty-five pregnancies in endometriosis group and 741 pregnancies in control group were included. Women with endometriosis had a higher risk of preterm delivery < 34 weeks (6.4% vs 2.8%, OR 2.42, 95% CI 1.22–4.82), preterm delivery < 37 weeks (17.8% vs 9.7%, OR 1.98, 95% CI 1.23–3.19), and neonatal admission to Intensive Care Unit (14.1% vs 7.0%, OR 2.04, 95% CI 1.23–3.36). At post hoc analysis, women with endometriosis and severe adenomyosis had an increased risk of placenta previa (23.1% vs 1.8%, OR 16.68, 95% CI 3.49–79.71), cesarean delivery (84.6% vs 38.9%, OR 8.03, 95% CI 1.69–38.25) and preterm delivery < 34 weeks (23.1% vs 5.7%, OR 5.52, 95% CI 1.38–22.09).

**Conclusion:**

Women with endometriosis who conceive naturally have increased risk of preterm delivery and neonatal admission to intensive care unit. When severe adenomyosis is coexistent with endometriosis, women may be at increased risk of placenta previa and cesarean delivery.

**Trial registration:**

Clinical trial registration number: NCT03354793.

## Introduction

Endometriosis during pregnancy has traditionally been considered to remain quiescent, due to the very high serum progesterone levels [1]. Nevertheless, a growing body of evidence suggests that women affected by endometriosis experience an unfavorable obstetric outcome as compared to the general population [2–5]. However, evidence from previous studies evaluating obstetric complications in women with endometriosis such as miscarriage, placenta previa, preterm delivery, cesarean delivery, preeclampsia, IntraUterine Growth Restriction (IUGR) and admission to Neonatal Intensive Care Unit (NICU), has been inconsistent [6]. Moreover, numerous studies have included women who conceived by means of Assisted Reproductive Technology (ART), although ART may in-itself increase the risk of obstetric complications. Other studies failed to adequately report the severity of endometriosis or to adjust the results for relevant characteristics such as age and parity [7].

Different authors have focused their attention on women with deep endometriosis only. However, although a specific association between rectovaginal endometriosis and placenta previa has been observed [8, 9], it has not been demonstrated that an anatomically more severe disease is associated with a worse obstetric outcome. Moreover, it has not been demonstrated that previous surgical treatment is able to prevent or reduce the prevalence of obstetric complications of endometriosis [10]. Therefore, we sought to evaluate the outcome of naturally conceived pregnancies managed according to standard obstetric care throughout the Italian territory, among women with endometriosis managed according to homogeneously adopted criteria in referral Centers and selected consecutively regardless the severity and the previous medical versus surgical treatment of the endometriotic disease.

## Materials and methods

The study was performed in 11 Italian Centers located in the cities of Bologna, Cagliari, Catanzaro, Firenze, Milano (three Hospitals), Palermo, Roma, Siena and Verona, between January 1st, 2017 and December 31st, 2018. All Hospitals are affiliated to the Endometriosis Treatment Italian Club (ETIC), an association gathering physician from tertiary referral Centers for endometriosis in Italy who share the same approach to the disease, including accurate ultrasonographic mapping, systematic use of medical therapy, radical surgical treatment with homogeneous indications and technique. The scientific references for our clinical approach are reported elsewhere [11–14].

The Institutional Review Board of the Promoting Center Fondazione IRCCS Cà Granda Ospedale Maggiore Policlinico of Milan approved the study (Comitato Etico Milano Area B; determination no. 732/2016). Subsequently, the study was approved by the local Institutional Review Board of each participating Center. Every woman signed an informed consent form before enrollment.

The Endometriosis Group included all consecutive women with either a surgical or nonsurgical diagnosis of endometriosis undergoing a follow-up examination during the study period in any of the eleven recruiting Centers. Nonsurgical diagnoses were based on ultrasonographic criteria in women with ovarian endometriomas; on visual inspection of the posterior fornix and biopsy of vaginal lesions in women with rectovaginal endometriosis; on ultrasonographic criteria, cystoscopic findings, and biopsy of vesical lesions in women with bladder detrusor endometriosis; on physical signs at rectovaginal examination and ultrasonographic criteria in those with deep lesions infiltrating the Douglas pouch and parametria; and on ultrasonographic criteria, double contrast barium enema, and rectosigmoidoscopy/colonoscopy findings in women with full-thickness bowel lesions. The Control Group included asymptomatic women attending outpatient clinics for periodical gynaecological care, contraception, or cervical cancer screening program, without a previous clinical or surgical diagnosis of endometriosis. Clinical evaluation in Control Group included pelvic bimanual examination in all women and additional diagnostic procedures such as, but not limited to, transvaginal ultrasonography when deemed appropriate.

All women in both Endometriosis Group and Control Group were invited to fill in a questionnaire on the outcome of their previous natural pregnancies. Most women filled in the questionnaire during the follow-up visit as outpatients, with the possibility of asking questions to the physician in case anything was not clear. In a minority of cases, the answers to the questionnaire were obtained by email or by telephonic interview. To obtain accurate answers, the questionnaire on obstetric outcome contained simple questions addressing major outcomes only and was presented only to women not older than 50 years, so that the time elapsed since their pregnancies was not too long. Women not having a positive recall about the questions they were asked were excluded from the study.

Among women in the Endometriosis Group, only the first pregnancy after the diagnosis of endometriosis was considered for inclusion in the study. Among multiparous women in the control group, first, second and third pregnancies were included.

Clinical data on women in the Endometriosis Group were collected from hospital charts. Rectovaginal endometriosis was defined as a nodule measuring at least 10 mm and determining adhesions between the sigmoid and/or rectum and the posterior aspect of the uterus. Isolated peritoneal lesions were represented by uterosacral endometriosis or endometriotic superficial lesions associated with adnexal adhesions; in no cases women with isolated superficial peritoneal spots incidentally visualized during surgery for other indications were included in the study.

Diagnosis of adenomyosis was based on the ultrasonographic detection of previously described reliable morphological markers such as asymmetrical myometrial thickening, myometrial cysts, linear striations, hyperechoic islands [15]. Data on irregularity and thickening of endometrial–myometrial junction zone were not systematically available so this marker was not included in the analysis. Diffuse severe adenomyosis was defined as involving more than 50% of myometrial surface and with a wall thickness ≥ 30 mm in at least two separated myometrial areas or as a globally enlarged uterus. Diffuse mild adenomyosis was diagnosed if those criteria for severe adenomyosis were not met.

Exclusion criteria were as follows: pregnancy achieved by ART; age > 50 years; not positive recall of previous pregnancies; voluntary termination of pregnancy; biochemical pregnancy; and increased risk of obstetrical complications: anti-phospholipid antibody syndrome, pre-gestational hypertension, history of recurrent abortions, uncorrected uterine malformations, twin pregnancy.

## Sample size

The sample size was calculated considering two main outcomes, the miscarriage rate and the rate of preterm delivery. On these bases, we aimed at about 300 pregnancies per group. Specifically, the expected rate of miscarriage in women without endometriosis is 20% [16]. When considering clinically relevant for women with endometriosis a 50% increase, i.e., a miscarriage rate of 30%, with a 5% level of significance and a power of 90% of a unilateral test, the sample size is 319 pregnancies in each group. Among women who deliver an alive neonate, the expected rate of preterm delivery in women without endometriosis is 10% [17]. When considering clinically relevant for women with endometriosis a 100% increase, i.e., a preterm delivery rate of 20%, with a 5% level of significance and a power of 90% of a unilateral test, the sample size is 216 pregnancies in each group.

## Statistical analysis

The characteristics of the study population (race, age, parity, BMI, adenomyosis, type of endometriosis) were summarized by means of number and percentage of subjects in the two groups and in the whole sample. Mean and standard deviation were also computed for numerical variables.

When evaluating women in the control Group who contributed more than one pregnancy, data clustering was accounted for in the analyses. Marginal logistic regression models were fitted to evaluate the impact of endometriosis on different pregnancy outcomes and complications. A different model was fitted for each of the following response variables: miscarriage in the first trimester, ectopic pregnancy, spontaneous abortion in the second trimester, termination of pregnancy in the second trimester, intrauterine fetal death in the third trimester, alive neonate delivered, placenta previa, preterm delivery < 37 week, preterm delivery < 34 week, admission to Neonatal Intensive Care Unit (NICU), cesarean delivery, IntraUterine Growth Restriction (IUGR), preeclampsia. In each model, the main explanatory variable was the presence or absence of endometriosis and the following adjusting factors were increasingly added into the model if the number of events would be sufficiently high (10 events for each variable): age group (≤ 30 years; 31–34 years; ≥ 35 years), parity (first, second pregnancy), BMI (< 25; ≥ 25), previous myomectomy (no, yes), uterine fibroids (no, yes).

A post hoc analysis was carried out within the endometriosis group on pregnancies ended with an alive neonate delivered, to compare the obstetric outcomes in women with severe adenomyosis and women with mild or absent adenomyosis. Different marginal logistic models were fitted taking into account the correlation of measure on the same women and considering the following obstetric outcomes as response variables: placenta previa, preterm delivery < 37 week, preterm delivery < 34 week, admission to Neonatal Intensive Care Unit (NICU), cesarean section, IntraUterine Growth Restriction (IUGR), preeclampsia. In each model, the main explanatory variable was the presence or absence of severe adenomyosis and the following adjusting factors were increasingly added into the model if the number of events would be sufficiently high (10 events for each variable): age group (≤ 30 years; 31–34 years; ≥ 35 years), parity (first, second pregnancy), bmi (< 25; ≥ 25).

The results were provided in terms of adjusted Odds Ratio (OR), with pertinent 95% Confidence Intervals (95% CI) and p-values. The adjusted OR and CI were not provided when one of the two study groups had no events in the response variable. The adjusted OR was considered statistically significantly different from 1 (the value indicating absence of association) when the corresponding p-value was less than 0.05.

All analysis were performed using R software, version 4.0.3, with package geepack, version 3.1–1, added.

## Results

We included in the study 453 women in the endometriosis group (one pregnancy for each woman only) and 794 pregnancies among 413 women in the control group. We excluded from further analysis 98 women in the endometriosis group because their only pregnancy had been achieved before the diagnosis of endometriosis and 53 pregnancies in the control group because of missing data. Overall, 1096 pregnancies were included in the study: 355 in the endometriosis group and 741 in the control group.

Table 1 shows the characteristics of the populations in the two groups. Mean age (SD) was 32.6 (4.4) years in the endometriosis group and 31.1 (5.5) years in the control group. Caucasian women were 311 (97.5%) in the endometriosis group and 382 (99.9%) in the control group. In the endometriosis group, the prevalence of ovarian disease was 72.9%, the prevalence of deeply invasive endometriosis was 40.8% and the coexistence of these two locations was observed in 24.2% of women. The prevalence of adenomyosis was 23.7% in the endometriosis group and 0.4% in the control group. Second pregnancies were 48 (13.5%) in the endometriosis group and 234 (31.6%) in the control group, third pregnancies were 4 (1.1%) in the endometriosis group and 94 (12.7%) in the control group. Women in the endometriosis group, as compared to the control group, had a lower prevalence of BMI ≥ 25 (13.5% vs 31.6%) and of non-operated uterine myomas (2.9% vs 7.5%) and a slightly lower prevalence of previous myomectomy (4.8% vs 6%).

**Table 1:** Characteristics of pregnancies in the study population

	Endometriosis group *n* = 355	Control group *n* = 741	Total *n* = 1096
Age			
≤ 30 years	103 (29.01%)	342 (46.15%)	445 (40.6%)
31–34 years	126 (35.49%)	194 (26.18%)	320 (29.2%)
≥ 35 years	126 (35.49%)	205 (27.67%)	331 (30.2%)
Endometriosis			
Ovarian	173 (48.7%)	NA	173 (48.7%)
Deep + ovarian	86 (24.2%)	NA	86 (24.2%)
Deep	59 (16.6%)	NA	59 (16.6%)
Peritoneal	19 (5.4%)	NA	19 (5.4%)
Adenomyosis			
Focal or diffuse mild	67 (18.9%)	3 (0.41%)	70 (6.41%)
Diffuse severe	17 (4.8%)	0 (0%)	17 (1.56%)
Multiparous	52 (14.65%)	328 (44.27%)	380 (34.67)
BMI ≥ 25	48 (13.52%)	234 (31.58%)	282 (25.73%)
Uterine myomas	10 (2.92%)	52 (7.53%)	62 (6%)
Previous myomectomy	16 (4.8%)	40 (6%)	56 (5.6%)

Table 2 shows the pregnancy outcome in the two groups. Pregnancies that did not end with the delivery of an alive neonate were 16.3% in the endometriosis group and 19.4% in control group. The risk of miscarriage was not significantly different between the endometriosis group and the control group. No significant differences between the two groups were observed also for the events of ectopic pregnancy, second trimester spontaneous abortion, second trimester termination of pregnancy for fetal anomalies and intrauterine fetal death.

**Table 2:** Pregnancy outcome in the endometriosis versus control two group

	Endometriosis *n* = 355	Controls *n* = 741	Total *n* = 1096	Adjusted odds ratio (95% CI)	*p* value
Miscarriage	50 (14.08%)	126 (17.00%)	176 (16.06%)	0.92 (0.59–1.43)	0.701
Ectopic pregnancy	2 (0.56%)	6 (0.81%)	8 (0.73%)	0.69 (0.14–3.46)	0.656
Spontaneous abortion	3 (0.85%)	9 (1.21%)	12 (1.09%)	0.69 (0.19–2.58)	0.584
TOP	0 (0.00%)	2 (0.27%)	2 (0.18%)	NA	NA
IUFD	3 (0.85%)	1 (0.13%)	4 (0.36%)	6.31 (0.65–60.85)	0.111
Alive neonate delivered	297 (83.66%)	597 (80.57%)	894 (81.57%)	1.13 (0.74–1.73)	0.573

Adjusted OR and IC were not computed because 0 event occurred in one of the 2 groups

Miscarriage: spontaneous abortion with sonographic crown–rump length < 13 weeks

Spontaneous abortion: intrauterine fetal death between 13 and 23 + 6 weeks

TOP: voluntary Termination Of Pregnancy for fetal anomalies (13–22 + 3 weeks)

IUFD: Intra Uterine Fetal Death > 24 weeks

*NA* not applicable

Among the 297 (83.7%) women in the endometriosis group who delivered an alive neonate, the prevalence and location of endometriosis [ovarian endometriosis 148 (49.8%), deep + ovarian endometriosis 72 (24.2%), deep endometriosis 45 (15.2%), peritoneal endometriosis 16 (5.4%)] as well as the prevalence and severity of adenomyosis [focal or diffuse mild 54 (18.2%), diffuse severe 13 (4.4%)] were comparable to those of the whole population of women with endometriosis. In this endometriosis-alive neonate delivered group, 30 (10.2%) women received hormonal treatment without surgical exploration, including 17 (5.8%) women with ovarian endometriosis, 11 (3.7%) women with rectovaginal endometriosis, one (0.3%) woman with vesical endometriosis and one (0.3%) woman with vaginal endometriosis. The remaining 264 (89.8%) women had previously undergone surgical treatment of endometriosis: 213 (72.4%) underwent stripping of mono- or bi-lateral ovarian cysts; 54 (18.4%) underwent shaving of a rectovaginal nodule; 34 (11.6%) underwent excision of nodules of the Douglas pouch and/or uterosacral ligaments without involvement of the bowel wall; 9 (3.1%) underwent excision of vaginal endometriosis; 6 (2.0%) underwent excision of ureteral endometriosis; 6 (2.0%) underwent segmental bladder resection; one (0.3%) underwent excision of a nodule involving the ciecum and appendix and 17 (5.8%) underwent excision of peritoneal endometriosis.

Table 3 shows obstetric complications in women in the endometriosis group (*n* = 297), as compared to women in the control group (*n* = 597) who delivered an alive neonate. Women with endometriosis had a significantly higher risk of preterm delivery before 37 weeks, preterm delivery before 34 weeks and neonatal admission to Intensive Care Unit. Cesarean delivery, placenta previa, IUGR and preeclampsia occurred more frequently in the endometriosis group, but the difference was not statistically significant.

**Table 3:** Obstetric complications in the endometriosis versus control two group

	Endometriosis *n* = 297	Controls *n* = 597	Total *n* = 894	Adjusted odds ratio (95% CI)	*p* value
Preterm delivery < 37w	53 (17.85%)	58 (9.72%)	111 (12.42%)	1.98 (1.23–3.19)	0.005
Preterm delivery < 34w	19 (6.40%)	17 (2.85%)	36 (4.03%)	2.42 (1.22–4.82)	0.012
Admission in NICU	42 (14.14%)	42 (7.04%)	84 (9.40%)	2.04 (1.23–3.36)	0.005
Placenta previa	8 (2.69%)	8 (1.34%)	16 (1.79%)	2.04 (0.76–5.49)	0.159
Cesarean delivery	122 (41.08%)	188 (31.49%)	310 (34.68%)	1.27 (0.89–1.80)	0.188
IUGR	12 (4.04%)	17 (2.85%)	29 (3.24%)	1.47 (0.69–3.11)	0.314
Preeclampsia	13 (4.38%)	23 (3.85%)	36 (4.03%)	1.21 (0.60–2.46)	0.597

*w* weeks of gestational age, *NICU* neonatal intensive care unit, *IUGR* intrauterine growth restriction

When excluding from the analysis women with placenta previa, women with endometriosis still presented a significantly higher risk of preterm delivery both before 37 weeks (16.3% vs 8.8%, OR 1.99, 95% CI 1.19–3.32; *p* = 0.008) and before 34 weeks (5.9% vs 2.5%, OR 2.52, 95% CI 1.22–5.17; *p* = 0.012).

The risk of neonatal admission to Intensive Care Unit remained significantly higher for women with endometriosis when excluding women who had a preterm delivery both before 37 weeks (11.1% vs 6.0%, adjusted OR 2.05.99, 95% CI 1.12–3.76; *p* = 0.020) and before 34 weeks (12.1% vs 7.1%, adjusted OR 1.87, 95% CI 1.08–3.24; *p* = 0.025).

Table 4 reports the post hoc analysis within the endometriosis group. Women with endometriosis and severe adenomyosis, as compared to women with endometriosis and mild or absent adenomyosis, had a significantly increased risk of placenta previa, preterm delivery before 34 weeks and cesarean delivery. They also had a higher risk of preterm delivery before 37 weeks, IUGR and preeclampsia, even though they were not statistically significant. The risk of neonatal admission to Intensive Care Unit was not significantly different in women with and without severe adenomyosis.

**Table 4:** Obstetric complications within the endometriosis group for women with severe diffuse adenomyosis versus diffuse mild or absent adenomyosis

	Adenomyosis severe *n* = 13	Adenomyosis mild or absent *n* = 283	Total *n* = 296	Adjusted odds ratio (95% CI)	*p* value
Placenta previa	3 (23.08%)	5 (1.77%)	8 (2.70%)	16.68 (3.49–79.71)	< 0.001
Cesarean delivery	11 (84.62%)	110 (38.87%)	121 (40.88%)	8.03 (1.69–38.25)	0.009
Preterm delivery < 34w	3 (23.08%)	16 (5.65%)	19 (6.42%)	5.52 (1.38–22.09)	0.016
Preterm delivery < 37w	5 (38.46%)	47 (16.61%)	52 (17.57%)	3.16 (0.88–11.34)	0.078
Admission in NICU	2 (15.38%)	40 (14.13%)	42 (14.19%)	1.19 (0.24–5.89)	0.829
IUGR	1 (7.69%)	11 (3.89%)	12 (4.05%)	2.06 (0.25–17.29)	0.505
Preeclampsia	1 (7.69%)	11 (3.89%)	12 (4.05%)	2.06 (0.25–17.29)	0.505

*w* weeks of gestational age, *NICU* neonatal intensive care unit, *IUGR* intrauterine growth restriction

The risk of placenta previa was not significantly increased in women with rectovaginal endometriosis as compared to women with other forms of endometriosis (6.2% vs 1.7%, adjusted OR 3.69, 95% CI 0.90–15.18; *p* = 0.071).

## Discussion

In the present study, we evaluate the relationship between endometriosis and obstetric complications among all consecutive women with the disease presenting at the endometriosis clinics of the eleven participating centers. Because in previous studies, obstetrical complications have been observed in women with all stages of endometriosis and a different impact of previous surgical versus hormonal treatment on obstetric outcome has not been demonstrated, we included women with any form of endometriosis and any previous treatment. The management of endometriosis was performed according to shared diagnostic and therapeutic protocols adopted by all centers.

A consequence of our enrolling criteria, i.e., obtaining obstetric information through a questionnaire rather than through Hospital records, was that we included in the study many women who delivered at their local Hospitals rather than in one of the recruiting centers. Therefore, our data may be generalized as reflecting average obstetric care for women with endometriosis in Italy. Moreover, since most of the eleven centers are also large University maternity hospitals that manage complicated pregnancy and delivery, we believe that the design of our study allowed minimizing the risk of missing uncomplicated pregnancies, thus avoiding a possible overestimation of obstetric complications both in women with and without endometriosis. The methods and statistics of the study were designed aiming to minimize clinical bias: the inclusion of natural pregnancies only allows a better evaluation of the impact of endometriosis on pregnancy outcome, without the confounding factor represented by in vitro fertilization and embryo transfer, that has been reported to be associated per se to a higher risk of adverse obstetric outcomes, such as preeclampsia, preterm delivery and low birth weight [18, 19]; finally, data were adjusted for clinical variables that may influence obstetric outcome such as age, parity, BMI, previous myomectomy and presence of uterine fibroids.

A limitation of the present study may be the inclusion of women with endometriosis that were at their second pregnancy (those in whom the diagnosis of endometriosis was established between the first and the second pregnancy) and thus, since endometriosis may be associates with infertility, the possible selection of a population with a better “a priori” obstetric prognosis as compared to the general population of women with endometriosis. Another limitation of the present study may be represented by the use of a questionnaire for collecting information on previous pregnancies, thus relying on women’s recall. However, women without positive recall of their previous pregnancies were not included in the study. Finally, the statistical power of our study was not adequate to stratify the results according to the different phenotypes of endometriosis, i.e., deep, ovarian or peritoneal, nor to detect differences in obstetrical outcome between surgically [20] and medically treated women.

Because adenomyosis, frequently associated with endometriosis, has been associated with obstetric complications such as preterm delivery and cesarean delivery [21], we sought to carry out a post hoc analysis evaluating the impact of this condition among women with endometriosis. A limitation of this analysis is that, due to the lack of systematic use of ultrasonographic scoring systems at the time of diagnosis, the assessment of the severity of adenomyosis relied greatly on the subjective evaluation of the examining physician and the only possible discrimination was between mild or severe disease. However, the sonographers responsible for the endometriosis clinic in each center were dedicated to the diagnosis of endometriosis and adenomyosis since no less than 10 years and the sonographic criteria were the same in each Center [15]. Importantly, moreover, post hoc analysis was conducted on severe adenomyosis only, the sonographic diagnosis of whom is the easiest and does not necessarily require a detailed scoring system. The observation that adenomyosis was virtually absent in the control group may be an underestimation due to a clinical bias. In fact, in some cases minimal or mild adenomyosis may have been overlooked or not reported because not deemed clinically relevant when evaluating asymptomatic women, especially multiparous, at routine ultrasonography.

Women with endometriosis did not show a higher risk of miscarriage as compared to the Control Group. In our series, miscarriage rate of 14% was slightly lower than that reported in previous studies, ranging between 18 and 21% [9, 22, 23]. To our knowledge, only three studies evaluated natural conceptions only and adjusted the results according to patient’s age, which is crucial for the estimation of the risk of miscarriage [9, 22, 23]. These three studies, in agreement with our findings, did not report an increased risk of miscarriage in women with endometriosis.

Neonates born from women with endometriosis, as compared to neonates born from women in the Control Group, had a significantly higher rate of admission to Intensive Care Unit (14% vs 7%). This outcome has been poorly investigated by previous studies. Mekaru et al., among women with endometriosis and natural conception, reported a prevalence of neonatal admission to Intensive Care Unit of 18%; however, in their study, the difference was not significant as compared to women without endometriosis [23]. Interestingly, in our series, the prevalence of neonatal admission to Intensive Care Unit in the Endometriosis Group remained significantly higher than in the control group when excluding from the analysis severely preterm or preterm neonates (prevalence of admission to NICU was 12.1% for neonates born after 34 weeks and 11.1% for neonates born after 37 weeks). The possible association between endometriosis and increased risk of neonatal admission to NICU requires further investigation.

We found a higher prevalence of preterm delivery < 37 weeks and of very preterm delivery < 34 weeks in women with endometriosis, confirming the findings of two meta-analyses evaluating this outcome among women with natural conception. Overall, the prevalence of preterm delivery in our series was 18%, higher than the 7% reported by the two meta-analyses [3, 24]. Moreover, the association with preterm delivery < 37 weeks and very preterm delivery < 34 weeks was still significant when excluding women with placenta previa.

Women with endometriosis did not have a significantly higher risk of placenta previa (2.7% as compared to 1.3% in women without endometriosis). This finding is in disagreement with a meta-analysis including natural pregnancies only, reporting a significantly higher prevalence of placenta previa among women with endometriosis as compared to women without endometriosis (5% vs 0.9%) [3]. Moreover, at odds with previous findings [9], the prevalence of placenta previa in our series was not significantly higher in women with rectovaginal endometriosis as compared to women with other forms of the disease (6.2% vs 1.7%). In comparison, among women surgically treated for rectovaginal endometriosis before pregnancy, a prevalence of placenta previa of 6.5% after radical excision [10] and of 17.8% after non-radical excision [8] has been reported. A tentative explanation for these observations may be that rectovaginal endometriosis was less severe in our series as compared to previous studies: in fact, women with rectovaginal endometriosis responded to hormonal treatment and did not require surgery in 16.9% of cases and none of those who underwent surgery required a segmental bowel resection. Another tentative explanation maybe that in the present study, at odds with previous studies, we included women previously treated for endometriosis who did not present any obstetric complication during pregnancy and therefore delivered at their local Hospitals without being referred to a third level maternity Hospital.

Women with endometriosis in our series did not have a significantly higher risk of cesarean delivery. This is in disagreement with previous findings [3, 5, 25]. The tentative explanations exposed in the above paragraph, i.e., a population with a less severe disease, could be adduced also for the non-increased rate of cesarean delivery in women with endometriosis. As for the possibility that our population of women with endometriosis is characterized by a better “a priori” obstetric prognosis due to the inclusion of women at their second pregnancy, it does not seem to explain a lower than expected prevalence of cesarean delivery. In fact, among women with endometriosis at their second pregnancy, the rate of previous cesarean delivery was high, i.e., 40%, with a consequent possibly high rate of repeated cesarean delivery. Unfortunately, since the indication for cesarean delivery was not asked for in the obstetric questionnaire of our study, due to the expected difficulty of the women in reporting this information correctly, we cannot comment on the indications for cesarean delivery in our series.

For the outcomes of preeclampsia and IUGR, we did not observe a significantly higher risk among women with endometriosis as compared to women without endometriosis. These figures are in agreement with two previous meta-analysis on women who conceived naturally [24, 26].

In the present study, we could not compare the obstetrical outcome for women with adenomyosis between the endometriosis group and the control group, because of the extremely low prevalence of adenomyosis in the control group. Therefore, to evaluate the possible role of this condition in determining obstetrical complications, as suggested in previous studies, we sought to perform a post hoc analysis comparing, within the group of women with endometriosis, the obstetrical complications of those with severe adenomyosis vs those with mild or absent adenomyosis. Interestingly, we found that women in the former group had a significantly higher risk of placenta previa and cesarean delivery. Although based on a small number of cases, these data seem to suggest that severe adenomyosis plays a significant role in increasing the risk of placenta previa and cesarean delivery in women with endometriosis.

In conclusion, our study may reflect the outcome of standard obstetric care in Italy, including women delivering at local rather than University Hospitals, among the overall population of women with endometriosis previously diagnosed and treated in referral Centers for the endometriotic disease. Our findings suggest that endometriosis is associated with an increased risk of preterm delivery and neonatal admission to intensive care unit. Further studies are needed to confirm our preliminary data showing an association between severe adenomyosis, coexistent with endometriosis, and placenta previa and cesarean delivery.
